# APOE2 orchestrated differences in transcriptomic and lipidomic profiles of postmortem AD brain

**DOI:** 10.1186/s13195-019-0558-0

**Published:** 2019-12-30

**Authors:** Iliya Lefterov, Cody M. Wolfe, Nicholas F. Fitz, Kyong Nyon Nam, Florent Letronne, Richard J. Biedrzycki, Julia Kofler, Xianlin Han, Jianing Wang, Jonathan Schug, Radosveta Koldamova

**Affiliations:** 1 Department of Environmental and Occupational Health, University of Pittsburgh, 130 De Soto Str., Pittsburgh, PA 15261 USA; 2 Department of Pathology, University of Pittsburgh School of Medicine, Pittsburgh, PA 15219 USA; 3 Department of Medicine & Biochemistry, Barshop Institute for Longevity and Aging Studies, UT Health-San Antonio, San Antonio, TX 78229 USA; 4 Department of Genetics, Functional Genomics Core, University of Pennsylvania, Philadelphia, PA 19104 USA

**Keywords:** Alzheimer’s disease, Human brain, APOE, RNA-seq transcriptomics, Shotgun lipidomics, Multi-omics analysis, Intracellular homeostasis and proteostasis

## Abstract

**Background:**

The application of advanced sequencing technologies and improved mass-spectrometry platforms revealed significant changes in gene expression and lipids in Alzheimer’s disease (AD) brain. The results so far have prompted further research using “multi-omics” approaches. These approaches become particularly relevant, considering the inheritance of *APOEε4* allele as a major genetic risk factor of AD, disease protective effect of *APOEε2* allele, and a major role of APOE in brain lipid metabolism.

**Methods:**

Postmortem brain samples from inferior parietal lobule genotyped as *APOEε2/c* (*APOEε2*/carriers), *APOEε3/3*, and *APOEε4/c (APOEε4/*carriers), age- and gender-matched, were used to reveal *APOE* allele-associated changes in transcriptomes and lipidomes. Differential gene expression and co-expression network analyses were applied to identify up- and downregulated Gene Ontology (GO) terms and pathways for correlation to lipidomics data.

**Results:**

Significantly affected GO terms and pathways were determined based on the comparisons of *APOEε2/c* datasets to those of *APOEε3/3* and *APOEε4/c* brain samples. The analysis of lists of genes in highly correlated network modules and of those differentially expressed demonstrated significant enrichment in GO terms associated with genes involved in intracellular proteasomal and lysosomal degradation of proteins, protein aggregates and organelles, ER stress, and response to unfolded protein, as well as mitochondrial function, electron transport, and ATP synthesis. Small nucleolar RNA coding units important for posttranscriptional modification of mRNA and therefore translation and protein synthesis were upregulated in *APOEε2/c* brain samples compared to both *APOEε3/3* and *APOEε4/c*. The analysis of lipidomics datasets revealed significant changes in ten major lipid classes (exclusively a decrease in *APOEε4/c* samples), most notably non-bilayer-forming phosphatidylethanolamine and phosphatidic acid, as well as mitochondrial membrane-forming lipids.

**Conclusions:**

The results of this study, despite the advanced stage of AD, point to the significant differences in postmortem brain transcriptomes and lipidomes, suggesting *APOE* allele associated differences in pathogenic mechanisms. Correlations within and between lipidomes and transcriptomes indicate coordinated effects of changes in the proteasomal system and autophagy—canonical and selective, facilitating intracellular degradation, protein entry into ER, response to ER stress, nucleolar modifications of mRNA, and likely myelination in *APOEε2/c* brains. Additional research and a better knowledge of the molecular mechanisms of proteostasis in the early stages of AD are required to develop more effective diagnostic approaches and eventually efficient therapeutic strategies.

## Background

The inheritance of *APOEε4* allele is the major genetic risk factor for late-onset AD [1]. The 3 alleles of human *APOE*—*APOEε2*, *APOEε3*, and *APOEε4*—translate to 3 protein isoforms, APOE2, APOE3, and APOE4, which differ only in 2 amino acid residues at positions 112 and 158 [2]. APOE is a 299-amino acid-long protein and a major component of low-density (LDL) and very low-density (VLDL) lipoproteins circulating in the blood. APOE is highly expressed in the brain, is secreted primarily by astrocytes, and its major role is to transport cholesterol and phospholipids as HDL-like particles in the interstitial fluid [3]. The first and major regulatory step in the formation of brain HDL is the lipidation of APOE by ATP-binding cassette transporter A1 (ABCA1) [4]. Properly lipidated APOE containing lipid particles in the brain affect synaptogenesis, play an important role in binding Aβ and lipid species, and facilitate their clearance through the blood-brain barrier and by microglia (reviewed in [4, 5]).

An increased risk for AD in *APOEε4* carriers is undisputable: it is materialized in the earlier age of AD onset (approximately half of ε4-homozygotes will develop AD before age of 85, compared to only 10% of non-carriers), accelerated course of the disease, and more pronounced brain pathology [6–8]. The molecular mechanisms mediated by *APOEε4* expression remain poorly understood, but a role for APOE4 in greater Aβ aggregation/deposition and neuronal toxicity, reduced clearance, and isoform-specific effects on neuroinflammation and neurogenesis have been demonstrated [5, 9]. The protective effect associated with *APOEε2* is far from understood and ironically, compared to *APOEε3/4* or *APOEε4/4*, *APOEε2/2*, and *APOEε2/3* genotypes, *APOEε2/c* are less represented in experimental and clinical studies. It is well established, however, that, excluding “oldest-old,” in the presence of clinical dementia and neuropathological criteria for AD, the effect of *APOEε2* is unaffected by age, it is independently associated with lower Braak neurofibrillary tangle stages, possibly fewer neuritic plaques, milder AD pathology, and less severe antemortem cognitive impairment [10–13].

During the last decade, advanced sequencing technologies, improved mass-spectrometry platforms, and “omics” approaches have been constantly providing massive datasets comprising tens of thousands of genes, metabolites, and lipid molecular species with enormous potential to address questions relevant to disease pathogenesis and development, and possibly, drug discovery for neurodegenerative disorders [14–20]. In this regard, the established association between lipid metabolism, Aβ generation, and its clearance from the brain [21], as well as recent reports on the changes in transcriptomic profiles in the brain of AD patients and AD model mice [22], has prompted further research using “multi-omics” assays. Their application is also motivated by the increasing evidence that changes in cholesterol and bilayer- and non-bilayer-forming phospholipids’ content play a role in the pathogenesis and progression of AD [5]. The “multi-omics” approaches become particularly relevant considering the inheritance of the *APOEε4* allele as a major genetic risk factor of AD, earlier onset, and aggravated AD phenotype, as well as the protective effect of inherited *APOEε2* allele. Studies of brain lipidomes in AD model mice revealed alterations in phospholipid composition of the synaptic mitochondrial membranes, with cardiolipin (CL) content diminished during the early stages of pathology, connecting specific lipid changes to AD-like neurodegenerative process [22]. Changes in the intracellular content of phosphatidylethanolamine (PE) as well as changes in its synthesis and metabolism have been associated with AD and other neurodegenerative disorders [23, 24]. While the vast majority of lipidomics studies have compared lipidomes of AD brains to non-demented healthy controls, there have been no reports correlating changes in brain transcriptomics profiles to changes in lipid profiles, particularly in the context of *APOE* genotype [25–27]. Here, we present “multi-omics” profiling of postmortem AD brain samples from the inferior parietal lobule.

The inferior parietal lobule was chosen for two reasons: (1) neurofibrillary tangle formation occurs in a well-defined order, starting in the medial temporal lobe early in the disease and subsequently progressing towards the lateral temporal, parietal, prefrontal cortices, and finally the motor and sensory areas [28, 29]. By contrast, in the earlier stages of the disease, amyloid deposits first affect the posterior association cortices and inferior parietal cortex; the areas of the medial temporal lobe might then be affected, but it is not very common in the early stages [28, 30, 31]. Thus, the goal was to reveal differential changes in brain transcriptomes and lipidomes possibly associated with *APOE* genotype that favors a delayed neurofibrillary tangle formation and slower amyloid deposition; (2) morphological and histochemical studies have shown that the initiation and progression of AD-related destruction inversely recapitulate primarily the progress of cortical myelination [28]. In humans, myelination of axons in the prefrontal association areas and temporal and parietal lobes has the most protracted myelination which continues until the end of the sixth decade of human life. Late-myelinating neocortical areas at the same time are the most vulnerable to developing the pathognomonic lesions of AD consisting of neuritic plaques and neurofibrillary tangles [32–34] (for a detailed review and extensive list of references, see Bartzokis [35]). Longitudinal MRI data and high-throughput analysis studies, however, have provided evidence that initial, early signs of mild cognitive impairment (MCI), based on Clinical Dementia Rating, are associated with a similar rate of atrophy across all medial temporal lobe regions and inferior parietal lobule [36, 37]. Moreover, comparing individuals without a diagnosis of MCI or AD but with cognitive complaints or cognitive decline, studies demonstrated involvement—detectable atrophy of posterior parietal lobule, more specifically the angular gyrus [38, 39]. Very recently, a study examining the distribution and severity of tau-PET binding in cognitively normal adults with preclinical AD, as determined by positive β-amyloid PET, found that the precuneus and inferior parietal cortex were among the eight regions with the highest tau-PET binding. The findings were interpreted as consistent with preclinical involvement of the medial temporal lobe (MTL) and parietal lobe in AD [40]. It is not known, however, if there are *APOE* genotype-associated differences in transcriptional profiles in the inferior parietal lobule at those very early—almost impossible to investigate—or very late stages of the disease, brain samples available at the time of death, and if they can explain the differences in disease progression.

The results of our study demonstrate *APOE* allele-associated gene expression and lipid patterns at advanced stages of the disease. Weighted gene co-expression network analysis (WGCNA) revealed 14 co-expression network modules with a significant correlation to the *APOE* genotype. Utilizing Gene Ontology (GO) analysis with highly connected hub genes and lists of differentially expressed genes, we identified enriched GO terms associated with myelination, macroautophagy, regulation of macroautophagy, protein ubiquitination, and phosphatidylethanolamine biosynthetic process. The correlation between significantly changed lipid molecular species and differentially expressed genes indicated that differences in intracellular catabolic processes that deliver cytoplasmic components to lysosomes, as well as polyubiquitylation—implicated in proteasomal and lysosomal protein degradation—are among those underlying *APOE* allele-associated differences in AD pathology.

## Methods

### AD brain samples

All samples (Tables 1 and 2) were provided by the University of Pittsburgh Alzheimer’s Disease Research Center (ADRC) brain bank and the Sanders-Brown Center on Aging at the University of Kentucky. Braak staging was performed on Bielschowsky-stained sections [30]. *APOE* allelic polymorphism was determined by a PCR-based assay [41]. Gray matter samples of *APOEε2/3* (later in the text and figures, the genotype is marked as *APOEε2/c*), *APOEε3/3*, *APOEε3/4*, and *APOEε4/4* (later in the text and figures, the last two genotypes are marked as *APOEε4/c*) genotypes from the right inferior parietal lobule were dissected and used for further processing. Age matching was confirmed by one-way ANOVA. Postmortem intervals (PMI) ranged between 1 and 15 h, with no significant difference between the groups (analysis by ordinary one-way ANOVA, Table 1).

**Table 1:** AD case demographics and neuropathological characteristics for transcriptomics

	APOE	Age	Sex	Braak stage
APOE2 carriers	2/3	93	F	3
	2/3	92	F	6
	2/3	92	F	3
	2/3	90	F	2
	2/3	91	F	5
	2/3	96	M	5
	2/3	67	M	6
	2/3	94	M	3
Age ± SD (years)		89.38 ± 9.2		
PMI (range; mean) hours		1.4-3.3; 2.48		
APOE3/3	3/3	80	F	6
	3/3	87	F	6
	3/3	81	F	6
	3/3	91	F	6
	3/3	89	F	6
	3/3	80	F	6
	3/3	80	F	6
	3/3	86	M	6
	3/3	85	M	6
	3/3	83	M	6
	3/3	81	M	6
	3/3	92	M	6
Age ± SD (years)		84.5 ± 4.4		
PMI (range; mean) hours		2-9; 4.08		
APOE4 carriers	3/4	83	F	6
	3/4	80	F	6
	3/4	89	F	6
	3/4	88	F	6
	3/4	84	F	6
	3/4	89	F	6
	3/4	90	F	6
	3/4	87	F	6
	3/4	88	M	6
	3/4	79	M	6
	3/4	76	M	6
	3/4	82	M	6
	3/4	84	M	6
	3/4	91	M	6
	3/4	80	M	6
	3/4	79	M	6
	4/4	79	F	6
	4/4	80	F	6
	4/4	82	M	6
	4/4	82	M	6
	4/4	84	M	6
	4/4	84	M	6
Age ± SD (years)		83.6 ± 4.2		
PMI (range; mean) hours		1-15; 4035

**Table 2:** AD case demographics and neuropathological characteristics for lipidomics

	APOE	Age	Sex	Braak stage
APOE2 carriers	2/3	93	F	3
	2/3	92	F	6
	2/3	92	F	3
	2/3	90	F	2
	2/3	91	F	5
	2/3	96	M	5
	2/3	67	M	6
	2/3	94	M	3
Age ± SD (years)		89.38 ± 9.2		
PMI (range; mean) hours		1.4-3.3; 2.48		
APOE3/3	3/3	80	F	6
	3/3	87	F	6
	3/3	81	F	6
	3/3	91	F	6
	3/3	89	F	6
	3/3	80	F	6
	3/3	80	F	6
	3/3	86	M	6
	3/3	85	M	6
	3/3	83	M	6
	3/3	81	M	6
	3/3	92	M	6
Age ± SD (years)		84.5 ± 4.4		
PMI (range; mean) hours		2-9; 4.08		
APOE4 carriers	3/4	83	F	6
	3/4	80	F	6
	3/4	89	F	6
	3/4	88	F	6
	3/4	84	F	6
	3/4	89	F	6
	3/4	90	F	6
	3/4	87	F	6
	3/4	88	M	6
	3/4	79	M	6
	3/4	76	M	6
	3/4	82	M	6
	3/4	84	M	6
	3/4	91	M	6
	3/4	80	M	6
	3/4	79	M	6
	4/4	79	F	6
	4/4	80	F	6
	4/4	82	M	6
	4/4	82	M	6
	4/4	84	M	6
	4/4	84	M	6
Age ± SD (years)		83.6 ± 4.2		
PMI (range; mean) hours		1-15; 4035		

### RNA isolation, processing, and sequencing

RNA isolation and purification were performed using RNeasy mini kit (Qiagen). To increase sample purity, rRNA was removed with Ribo-Zero Gold rRNA Removal Kit (Illumina) and libraries were generated using mRNA Library Prep Reagent Set (Illumina) with the incorporation of barcodes for multiplexing. A targeted size selection was performed using Pippin Prep (Sage Science), the quality of the libraries was assessed on a 2100 Bioanalyzer (Agilent) and sequenced on Illumina HiSeq 2000 at the Functional Genomics Core, University of Pennsylvania, Philadelphia, PA.

### Weighted gene co-expression network analysis

Unsupervised hierarchical clustering and WGCNA were performed as previously [42–44], using sequencing datasets of 42 samples. The co-expression network was created with a raw count exclusion so that genes below 5 reads per million (RPM) mapped were removed to eliminate noise. Samples were clustered by gene expression profiles to identify the potential outliers. A scale-free topology model was applied, and a weighted network was constructed by Pearson correlation between all pairs of genes. Modules (functional networks) were detected using automatic block detection with a minimum module size of 20 and a merge height of module clustering for genes of 0.25. The dataset was adjusted for batch effects using an Empirical Bayes-moderated linear regression model which removes covariates potentially introduced due to variability between sequencing runs. The modules were assigned an arbitrary color then correlated with trait data—*APOE2/c*, *APOE3/3*, and *APOE4/c*. Within the modules, hub genes were identified by module membership (MM > 0.8), which is the connectivity between genes and a given module, and gene significance (GS > 0.2), which is the correlation between gene expression and *APOE* genotype. Modules for further analysis were selected only if their correlation within the expression network was significant (*p* < 0.05) and if the genes of a given module generated significant GO terms with false discovery rate (FDR) < 0.05.

### Differential gene expression analysis

For read mapping and summarization (human reference genome, hg38), we applied Subread (http://subread.sourceforge.net) averaging 15.2 million successfully aligned reads per library. Differential expression was analyzed using “edgeR” (http://www.bioconductor.org/packages/release/bioc/). To accommodate the experimental design, we applied a generalized linear model, and to account for gene-specific variability from both biological and technical sources, the working hypothesis was tested in a quasi-negative binomial framework [45]. The test for significant differential expression (DE) in each gene was performed by quasi-likelihood *F* test [45, 46]. Multiplicity correction was performed by applying the Benjamini-Hochberg method on *p* values, to control the FDR. The total number of DE genes, therefore, is a sum in each direction of *p* values at an FDR of 1%. Volcano plots were generated by comparing the genotypes with a calculated FDR and fold change (FC) for each gene. The calculated values were log-transformed (−log10 for FDR and log2 for FC) to generate the classic volcano shape of the data. Each point on the plots indicates a single gene, and genes that are significantly different (FDR < 0.05; −log10(0.05) = 1.3) between the groups are highlighted in red or blue depending on the direction of the alteration. Functional annotation of differentially expressed genes was performed using Database for Annotation, Visualization and Integrated Discovery (DAVID) (https://david.ncifcrf.gov).

### Lipidomics

Multi-dimensional mass spectrometry shotgun lipidomics (MDMS-SL) assays [18, 47, 48] were performed to determine the effects of *APOE* alleles on brain lipidome (demographics in Table 2). Brain samples were homogenized in PBS and protein content determined using the BCA protein assay kit (Pierce). Internal standards for measuring individual molecular species of the major lipid classes were added to the homogenates prior to lipid extraction. Lipid extraction was performed by the methyl-tert-butyl ether (MTBE) method, with resuspension in chloroform/methanol (1:1 v/v) solution and nitrogen flush. The samples were analyzed on a triple-quadrupole mass spectrometer (Thermo Fisher) equipped with an automated nanospray apparatus NanoMate and Xcalibur system [47]. Identification and quantification of all reported lipid molecular species were performed using an in-house automated software program [47].

### General data analysis

General statistical analyses and graphs were performed and presented using GraphPad Prism (v7) or R (v3.6.0). The results are reported as means ± SEM. Differences were considered significant when *p* < 0.05. Specific statistical and mathematical approaches are presented in the sections above. Detailed descriptions of those exist in the citations as indicated.

## Results

### *APOE* genotype is differentially associated with AD brain transcriptome

To determine the association of *APOE* alleles with gene expression, we performed RNA-seq using samples of the inferior parietal lobule of AD postmortem brains. We compared three groups/genotypes: *APOEε2/c*, *APOEε3/3*, and *APOEε4/c* (Table 1), age- and sex-matched. All of the samples were confirmed AD Braak stages 2–6. The analysis of PMI did not reveal any differences between the groups.

**Fig. 1:**
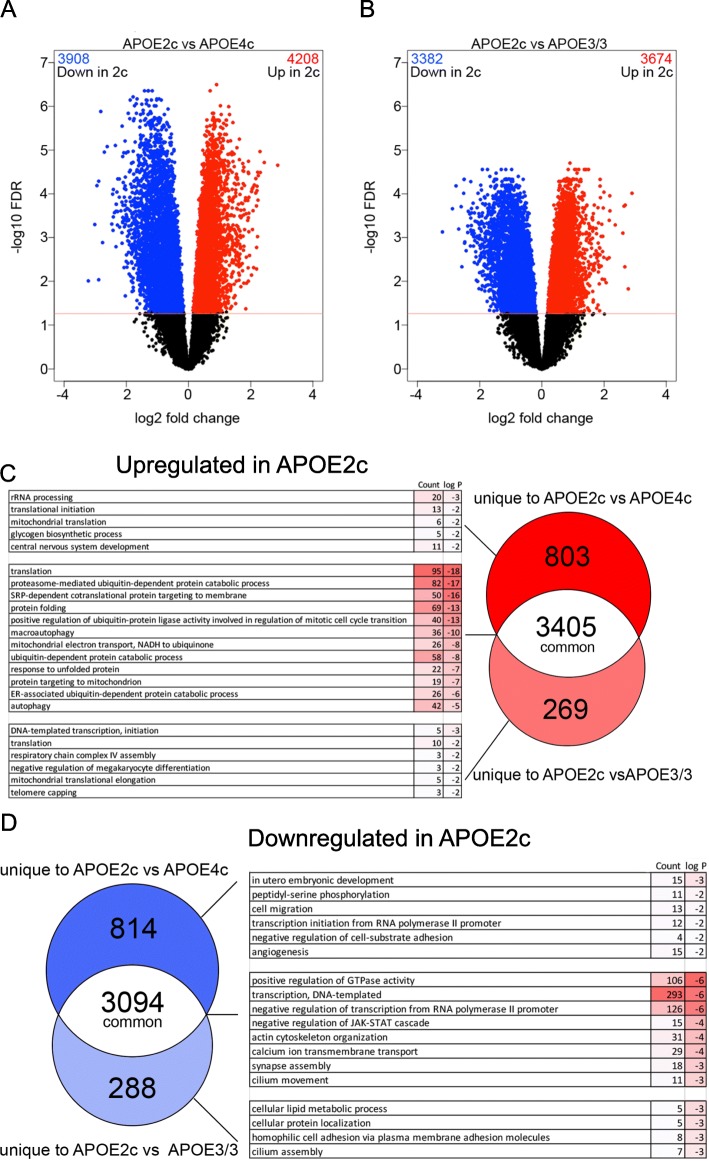
APOE genotype is differentially associated with brain transcriptome. RNA-seq datasets of *APOEε2/c* (*N* = 8), *APOEε3/3* (*N* = 12), and *APOEε4/c* (*N* = 22) samples were analyzed using edgeR. **a**, **b** Volcano plots representing the differentially expressed transcripts, colored in blue (downregulated) and red (upregulated) at FDR < 0.05. **c** Venn diagram with expanded GO terms generated from the genes that are upregulated in *APOEε2/c* vs *APOEε4/c* and *APOEε2/c* vs *APOEε3/3*. Shown are the number of genes that are upregulated in both comparisons (3405, white), genes uniquely upregulated in *APOEε2/c* vs *APOEε4/c* comparison (803, bright red), and genes uniquely upregulated in *APOEε2/c* vs *APOEε3/3* (269, coral). **d** Venn diagram showing the number of genes downregulated in both comparisons (3094, white), uniquely downregulated in *APOEε2/c* vs *APOEε4/c* comparison (814, dark blue), and genes uniquely downregulated in *APOEε2/c* vs *APOEε3/3* (288, light blue), as well as the associated GO terms for each group. Next to the Venn diagrams are the lists of the GO terms generated by DAVID using unique and common genes separately for each comparison

To determine differentially expressed genes, we used edgeR and analyzed the 3 groups simultaneously. Comparing *APOEε2/c* vs *APOEε4/c* (Fig. 1a) and *APOEε2/c* vs *APOEε3/3* (Fig. 1b), we identified a large number of significant, up- and downregulated transcripts at FDR < 0.05 cutoff. When *APOEε4/c* were compared to *APOEε3/3*, we did not find differentially expressed genes at FDR < 0.05. Within the first two comparisons, we identified 3405 genes that were commonly upregulated (in *APOEε2/c* vs *APOEε4/c* and *APOEε2/c* vs *APOEε3/3*; Fig. 1c—shown in white on the Venn diagram). Enrichment analysis revealed that common genes, with increased expression in *APOEε2/c*, clustered primarily in highly significant GO terms involved in translation, proteasome-mediated ubiquitin-dependent protein catabolic process, response to unfolded protein, signal recognition particle (SRP)-dependent protein targeting, endoplasmic reticulum (ER) translational translocation, ER stress response, autophagy, and mitochondrial electron transport. (Fig. 1c). The 3094 common downregulated genes of *APOEε2/c* samples clustered in GO terms representing positive regulation of GTPase activity, Ca ion transmembrane transport, actin cytoskeleton organization synapse assembly, and cilium movement (Fig. 1d).

### WGCNA identified modules of gene co-expression network that differentially correlated to *APOE* genotype

To analyze transcriptomic data of all samples and to reveal the gene co-expression network, we applied WGCNA using the methodology and statistical approaches previously described [43, 49, 50]. The WGCNA started out from 18,170 genes, and the identified modules of co-expressed genes were related to *APOE* genotypes and GO information. Since gene modules correspond to biological pathways, the analysis of modules and their highly connected intramodular hub genes amounts to a biologically meaningful data reduction scheme. Highly correlated module genes are represented and summarized by their first principal component, referred to as the module eigengene, or ME, which can be considered a representative of gene expression profiles in the module [51, 52]. The ME is used to quantify how close a gene is to a given module. Module definition in this study was based on the gene expression level in the inferior parietal lobule of 42 samples. Thus, module membership measures allowed annotation of all genes in the sequencing dataset (excluding those with an expression level indistinguishable from the sequencing noise) and screening for *APOE* genotype related intramodular hub genes. We used functional enrichment analysis to present the biological significance of the ME and to identify putative *APOE* genotype-associated pathways.

**Fig. 2:**
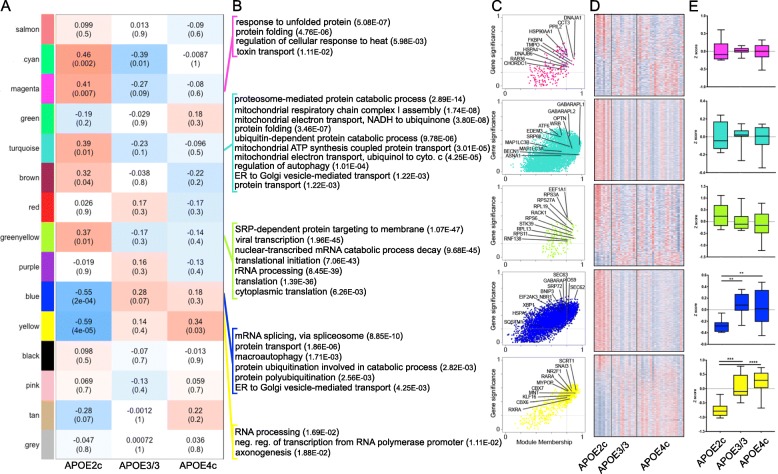
Gene co-expression network modules—correlation to APOE genotype and GO enrichment. WGCNA was applied to determine the correlation of module eigengenes (ME) to *APOE* allele combinations. **a** The relationship table shows the correlation between the module eigengene (rows) and genotype (columns) with Pearson correlation values and *p* values in parentheses. Red denotes a positive, and blue denotes a negative correlation. **b** Top GO terms (10 or less) generated from the genes associated with modules significantly impacted by *APOE* genotype (Benjamini correction for multiple comparisons, shown in parentheses). **c** Module membership (MM) vs gene significance (GS) plots for all genes within a given module. Genes above 0.8 MM and 0.2 GS are identified as hub genes, with genes of interest labeled on the plots. **d** Heatmaps of genes within modules’ *z*-scores with values ranging from 7 (red, above average) to − 7 (blue, below average). **e** Whisker plots of the average *z*-score within a module for each sample; min and max values are indicated with tails; the numbers of transcriptomes of each genotype are as on Table 1

First, using WGCNA, we correlated the networks of co-expressed module eigengenes—ME—to three traits, sex, age, and *APOE* genotype (Additional file 6: Figure S1). As visible, the age did not have a significant effect, and MEsalmon was the only module that correlated significantly to sex. This correlation of this module is driven primarily by genes important for sex determination such as *UTY* and *DDX3Y* located on the Y chromosome or *DDX3X* and *XIST* located on the X chromosome. There were no significant GO terms generated by the genes of this module. Second, since *APOEε2/c* genotype correlated significantly to the ME of seven modules, we analyzed their correlation to *APOEε3/3* and *APOEε4/c* genotypes (Fig. 2a and Additional file 1: Table S1). As shown, in addition to the eigengenes of the seven already mentioned modules with highly significant correlations with *APOEε2/c* genotype, there was only one of those significantly correlated with *APOEε4/c*. GO enrichment analysis for those modules was performed using DAVID (Fig. 2b). We identified hub genes as those with module membership (MM) above 0.8 and gene significance (GS) of 0.2 (Fig. 2c; highlighted are genes of interest within some of the GO terms. The calculated *z*-scores of the genes within the modules and the average *z*-score within each module of each sample are presented as a heatmap and whisker plots on Fig. 2d & e correspondingly).

MEmagenta correlated positively to *APOEε2/c* and negatively to APOE*ε3/3* and *APOEε4/c* and is enriched for GO terms related to protein folding and response to unfolded protein (Fig. 2b). MEgreenyellow was represented by GO terms associated with signal recognition particle (SRP)-dependent protein targeting and translational initiation. Functionally, very close to GO terms in MEgreenyellow were signal transduction pathways identified in MEturquoise—ubiquitin and proteasome-mediated protein catabolism, ER to Golgi vesicle-mediated transport, and protein folding. GO term regulation of autophagy and macroautophagy with differentially upregulated genes in *APOEε2/c* samples were identified in MEturquoise and MEblue. MEyellow was the only positively upregulated module in *APOEε4/c* samples, comprising GO term RNA processing, regulation of transcription from RNA polymerase promoter and axonogenesis. As seen from Additional file 1: Table S1, MEcyan is unique and consists entirely of genes coding for small nucleolar RNAs (snoRNA). Differentially expressed individual and clustered snoRNAs coding units are spread out across the entire genome and their host genes are unrelated. SnoRNA coding units are of both H/ACA and C/D boxes [53]. At FDR < 0.05, 22 SNORAs and SNORDs altogether were differentially upregulated in *APOEε2/c* vs *APOEε4/c*; 19 of those were upregulated in *APOEε2/c* vs *APOEε3/3*. These snoRNAs, however, did not generate any significant GO terms. There is no published information that any of those have been implicated, or associated in any way, with the pathogenesis of AD or other neurodegenerative disorders.

### In AD brain, *APOE* allele combinations are associated with distinct lipid profiles

**Fig. 3:**
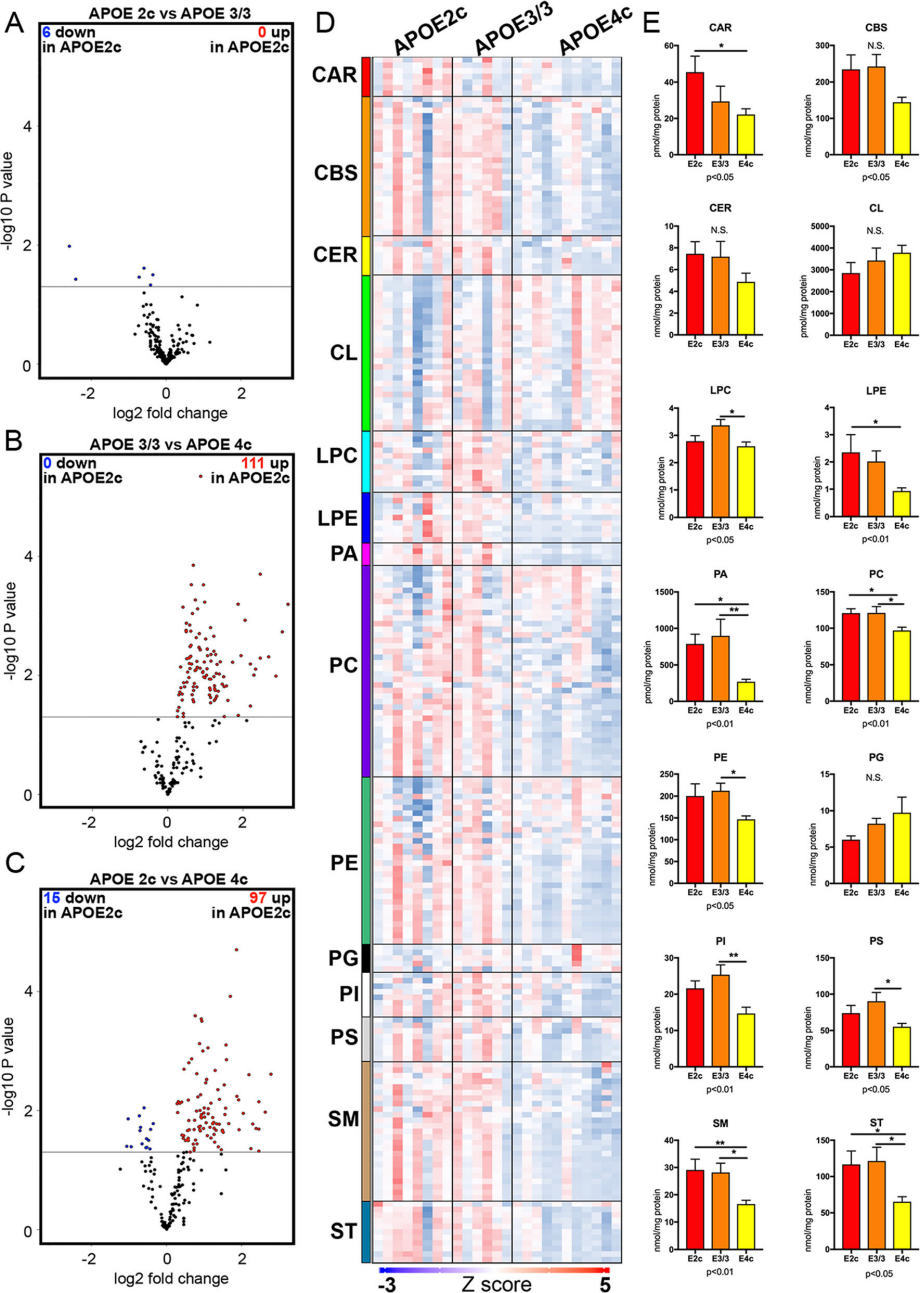
Association of APOE allele combinations with AD brain lipidome. MDMS-SL was performed to quantify 14 lipid classes and 216 molecular lipid species of inferior parietal lobule brain samples of *APOEε2/c* (*N* = 8), *APOEε3/3* (*N* = 6), and *APOEε4/c* (*N* = 11) genotypes. **a**–**c** Volcano plots show all 216 molecular lipid species quantified by MDMS-SL. Each point represents log2 fold change and −log10 *p* value of a particular lipid species. Significantly affected species at *p* < 0.05 cutoff are colored in blue (decreased) or red (increased). **d** Heatmap of all lipid subspecies of the 14 lipid classes. Each row in the heatmap represents unique lipid subspecies, denoted by lipid class code; within a class, rows are ordered by molecular mass; each column represents a sample. Data is presented as a *z*-score where red values are above average and blue values are below average. **e** Bar plots of the sum of all species within a lipid class. Statistics is by one-away ANOVA (*p* values shown at the bottom of each graph), followed by Tukey post hoc test (significant differences shown on the graph: *< 0.05, **< 0.01, N.S. no significance)

Because APOE is a major lipid transporter and the most important one in the brain, we analyzed the lipid composition of the inferior parietal lobule of AD brains of *APOEε2/c*, *APOEε3/3*, and *APOEε4/c* genotypes (demographics in Table 2). We applied shotgun lipidomics to measure the major phospholipid classes and their molecular species. The analysis identified 14 major lipid classes, comprising 215 molecular species. The differences between the lipid species are graphically presented in Fig. 3a–e. Significantly changed lipid species between genotypes are presented in 3 separate volcano plots (Fig. 3a–c). The heat map in Fig. 3d illustrates the level of each of the molecular species in each of the brain samples, and the comparison between the normalized total values of lipids in each of the lipid classes and genotypes is further illustrated by the bar plots in Fig. 3e (Additional file 2: Table S2; Additional file 3: Table S3, Additional file 4: Table S4, and Additional file 5: Table S5 for color codes and abbreviations). Ten of the lipid classes showed differences in their total normalized amounts in at least 1 of the comparisons, and in each of the comparisons, the levels of lipids in *APOEε4/c* were significantly lower. Moreover, PA, PC, PE, SM, and ST were significantly lower in *APOEε4/c* samples compared to either *APOEε2/c* or *APOEε3/3*. All of the mitochondrial membrane bilayer-forming phospholipids—PC, PS, PI, and 2 of the 3 non-bilayer-forming phospholipids, PE and PA, except CL—were significantly diminished in *APOEε4/c* samples. In a sharp contrast to transcriptomic profiles, the difference between *APOEε3/3* vs *APOEε4/c* brain lipidomes was very significant. Moreover, in 4 of the lipid classes—LPS, PE, PI, and PS—a highly significant difference was identified only between *APOEε3/3* vs *APOEε4/c* samples.

## Discussion

The goal of this study was to reveal and analyze a differential association of APOE genotype with transcriptomic and lipidomic profiles in postmortem AD brain samples and to determine correlations. Since *APOEε2* allele is significantly related to a reduced disease risk, especially in people under the age of 85 years [6–8], in groups with no statistical difference by age at death, we would expect *APOEε2/c* postmortem brains at lower Braak stages and not as severe brain pathology. Thus, transcriptomic profiling of *APOEε2/c*, *APOEε3/3*, and *APOEε4/c* postmortem brains would likely reveal changes associated with the corresponding *APOE* allele.

Our study provides RNA-seq and mass-spectrometry lipidomics data derived from the inferior parietal lobule of *APOEε2/c*, *APOEε3/3*, and *APOEε4/c* postmortem brains at known age of death and sex, at an advanced stage of AD, and allows interpretations in the context of gene expression and differences in brain lipidomes. We analyzed the changes in the gene expression using two different statistical approaches with their corresponding computational tools: WGCNA [51] with an initial normalization step executed by DESeq2 [54], and edgeR [45]. WGCNA builds gene co-expression networks and reveals the relationship between biologically meaningful modules based on all transcripts excluding those indistinguishable from the sequencing noise, in all samples; edgeR performs RNA-seq profiling and identifies differentially expressed (DE) genes and molecular pathways between two or more biological conditions. In our study, lists of genes that belonged to individual modules within the network—WGCNA—or identified as DE genes based on the comparisons between genotypes in edgeR were further processed to reveal GO terms and categories and to demonstrate differences between *APOE* genotypes.

We found that four of the significantly correlated modules of the network contained hub genes that are involved in GO terms with highly significant enrichment. The modules enclosed pathways with biological functions that are considered or suspected as impaired and associated with AD molecular pathology. In MEmagenta, MEgreenyellow, and MEturquoise modules, with highly positive correlations to the network, a number of GO terms remarkably overlapped with GO terms generated by genes found differentially upregulated by edgeR in *APOEε2/c* samples when compared to *APOEε3/3* and *APOEε4/c* (Figs. 1 and 2). These highly enriched GO terms were represented by pathways associated with proteostasis in ER, response to unfolded protein, intracellular protein, and organelle degradation—selective and basal autophagy, macroautophagy and its regulation, ubiquitination and ubiquitin-mediated proteasomal degradation, and SRP-dependent protein targeting.

Intracellular catabolic processes deliver cytoplasmic components to lysosomes through autophagic vacuoles. During the course of AD, autophagy and macroautophagy have a range of effects—deleterious as well as protective, depending on the stage of the pathologic process [55, 56]. In recent years, the results of research aiming at a better understanding of proteostasis in neurons have identified interrelated regulatory mechanisms and posttranslational modifications that are part of the ubiquitin proteasomal system and autophagy-lysosomal pathway, operating in concert to achieve intracellular protein balance [57]. Importantly, as discussed above, in a number of modules of the co-expression network, numerous highly significant GO terms are associated with macroautophagy, regulation of macroautophagy, protein ubiquitination, and proteasome-mediated ubiquitin-dependent catabolic process (Fig. 2b).

We found particularly interesting module MEcyan and the set of its genes—all snoRNAs. Functionally, box C/D and H/ACA snoRNAs play an important role in posttranscriptional modifications of mRNAs, impacting translational machinery and ultimately protein synthesis. C/D guide ribonucleoproteins to conduct the methylation of the 2′-OH group of ribose, while H/ACA rotate and convert C-5 ribosyl isomer of uridine into pseudouridine through a rotational break of C–C glycosidic bond and formation of an N–C one [53]. The most well-studied box C/D snoRNAs—SNORDs—are located in two large, imprinted gene clusters at human chromosome region 15q11q13 (the SNURF-SNRPN domain) and at 14q32 (the DLK1-DIO3 domain) [58]. They are expressed respectively only from the paternally and maternally inherited alleles. While there is evidence to consider the altered expression of SNORD115 and SNORD116, a primary cause of Prader-Willi syndrome, most recently those two and some other snoRNAs, has been implicated in the pathogenesis of schizophrenia [59–63]. If and how exactly SNORDs are involved in altered mRNA splicing in the pathogenesis of schizophrenia is not clear yet, but none of those has been so far associated with AD. The biology and function of box H/ACA snoRNAs—SNORAs—have been extensively studied [64], and their role in cancer is well established [65]. Studies addressing the role of SNORAs in AD and results of research so as to compare our findings are not available. The role of snoRNAs in the pathogenesis of AD, however, will evolve as an important research topic, and we believe further research will definitely reveal important aspects of their biogenesis, structure, and mechanisms implicated in the pathogenesis of the disease.

There were significant and consistent changes in the total amount of lipids and numerous individual molecular species in 10 of the 14 lipid classes analyzed in this study (Fig. 3). In all of those instances, there was a significant decrease of phospholipids in *APOEε4/c* vs either *APOEε2/c* or *APOEε3/3* or vs both genotypes, like in PA, PC, SM, and ST. While in agreement with previously published alterations/decrease of phospholipids during the course of pathogenic processes in AD [66], the differences between the lipidomes revealed in our study become particularly important since they can be correlated with the changes in the transcriptomic profiles of the exact same brain area. These correlations help to better understand the contribution of different *APOE* allelic combinations towards differences in the disease progression and possibly AD pathogenesis. In this regard, particularly relevant are the metabolic and regulatory pathways that are involved in the maintaining of a healthy cellular proteome, a process collectively called proteostasis, through highly coordinated intracellular protein and organelle degradation. A fundamental challenge in proteostasis is the protection against misfolded or damaged proteins and protein aggregates that severely disturb cellular functions. If we consider the most significant differences in the transcriptomic profiles associated with *APOE2/c* genotype vs *APOEε4/c* and *APOEε3/3*, we can link the enriched metabolic and regulatory pathways to the differences in proteostasis. Thus, we are suggesting a model explaining the protective effect of *APOEε2* allele in AD by the differences in some well-defined steps of the unfolded protein response, ER stress and ER-associated degradation (ERAD), and proteasomal and lysosomal intracellular degradation. We are assuming that transcriptional upregulation of genes, an important part of the pathways discussed below, facilitates sustained ER homeostasis that provides better protection against misfolded or damaged proteins and organelles. Such a model is supported by the following correlated *APOE* genotype-associated lipidomic profiles:

First, key genes in the pathways that target proteins to the ER are differentially upregulated in *APOEε2/c*: (a) in the co-translational translocation pathway *SRP68* and *SRP72*, encoding the subunits of the SRP; (b) *SEC61* (all three subunits of the heterotrimeric complex), *SEC62*, and *SEC63* at key regulatory steps of both co-translational translocation and SRP-independent posttranslational translocation pathways. Importantly, *SEC62* functions as a LC3-II receptor, and the interaction with LC3-II promotes the maintenance and recovery of ER homeostasis through clearance of select ER constituents by autolysosomes [67]; (c) within the pathway of tail-anchored proteins, gene orthologs *WRB* and *ASNA1* that target proteins to ER are significantly upregulated in *APOEε2/c* samples, too. Similarly, in ER stress pathways and unfolded protein response activation, genes coding for proteins in all three key axes—transcription factor *XBP1*, *HSPA5* (GRP ortholog), and *EIF2K3* (PERK ortholog), and transcription factor *ATF6*—are differentially upregulated in *APOEε2/c* AD samples; (d) a cellular pathway that recognizes unfolded/misfolded proteins in the ER and targets them for ubiquitination and subsequent degradation by the proteasome in the cytosol is called ERAD. Three of the key genes, *EDEM2*, *EDEM3*, and *OS9*, are upregulated in *APOEε2/c* samples. The genes are coding for proteins responsible for recognition of N-glycan structures, targeting and routing misfolded proteins for ubiquitination and subsequent degradation by the proteasome in the cytosol [68, 69].

Second, LC3-PE conjugation is an indispensable step for autophagy-related genes (ATG) to exert their function in autophagy, and for that reason, the availability of sufficient PE is critical, too. The first step of phagophore formation is the conjugation of PE to the mammalian orthologs of yeast ATG8/LC3. Five of those mammalian orthologs *MAP 1LC3A*, *MAP 1LC3B*, *GABARAP*, *GABARAPL1*, and *GABARAPL2* are upregulated in *APOEε2/c* brain samples. The subsequent generation of a covalent bond between ATG8 and PE requires a complex composed of ATG5-ATG12/ATG16L1; the genes of this complex are also upregulated in *APOEε2/c*.

Third, autophagy receptors (similarly to LC3-II/SEC62 complex) bind to cytosolic LC3 conjugated to PE and have a major role in selective autophagy, which is a process that regulates the abundance of specific cellular components [70]. Autophagy receptors target protein complexes, aggregates, and whole organelles into lysosomes. Selective autophagy pathways, named after the cargo—aggrephagy, mitophagy, xenophagy, and pexophagy—can be ubiquitin (Ub)-dependent and Ub-independent. Four autophagy receptors—*p62*, *NBR1*, *OPTN*, and *BNIP3*—that can act on one or several pathways were upregulated in *APOEε2/c* brain samples as common genes for both comparisons, against *APOEε3/3* and *APOEε4/c* (*p62* only in *APOEε2/c* vs *APOEε4/c*). Numerous upregulated genes involved in the proteasome-mediated Ub-dependent protein catabolic process were significantly upregulated in *APOEε2/c* brain samples, as well.

Fourth, *Beclin1* (*BECN1*), acting as a molecular platform assembling an interactome which regulates the initiation of the autophagosome, is upregulated in *APOEε2/c* brain samples. Although results from a previous study [71] that demonstrated decreases in Beclin1 levels in AD midfrontal cortex gray matter still remain to be confirmed [72, 73], numerous reports show the inhibition of Beclin1 interactome impairs autophagy and promotes AD-like pathology in in vitro and in vivo model systems [71, 74].

Fifth, but not least, autophagy is highly dependent on the proper lipidation through PE conjugation of several proteins critical for phagophore formation, elongation, and autophagosome generation [75–77]. Significantly lower amounts of PE in *APOEε4/c* brains likely provide conditions for less efficient initiation of autophagy [78, 79].

In the “Results” section, we indicated that the comparison of *APOEε3/3* vs *APOEε4/c* did not reveal differentially expressed genes at FDR < 0.05. While results of a study with a design and selection of groups as in our own have not been published so far, the differences in the expression profiles of *APOEε3/3* vs *APOEε4/4* and *APOEε3/4* (the latter two groups similar to our *APOEε4/c*) were a goal of a study published in 2007 by Xu et al. [27]. The study concluded that the expression pattern of APOE3/4 and APOE4/4 in the hippocampus of AD patients differed substantially from that of APOE3/3 AD patients. Since we have found no difference between the transcriptomic profiles of *APOEε4/c* and *APOEε3/3* brain samples, there is an obvious discrepancy. The technologies used for transcriptomic profiling in both studies—SAGE, Xu et al. [27], and NGS on Illumina platform, together with the methodology to analyze the differential gene expression—edgeR—in our study could be a reason for the differences; other explanations are possible as well: (1) stage of the disease—all our *APOEε4/c* samples are at advanced Braak stage 6 vs stages 3–4 for the samples in Xu et al.; (2) brain area used for transcriptomic profiling—the inferior parietal lobule in our case vs MTL in Xu et al. While WGCNA analysis after clustering within *APOEε4/c* group in our study was precluded by the insufficient number of samples, the questions raised by the discrepancy of the two studies should be addressed in the future, and hopefully, the answers would elucidate important aspects of the protective effect of *APOEε2* allele in AD.

The most recent study [80], addressing *APOE* genotype-associated differences in transcriptional profiles of postmortem AD samples, was published just a week before the submission of this article. While the most important difference with our study is the relative heterogeneity of their samples (combining traumatic brain injury and AD samples), the authors made very important conclusions that, to some extent, strongly support the results we are presenting here: regardless of the sex, the observed difference in transcription patterns for all brain regions analyzed including parietal cortex significantly correlated to the presence or absence of *APOE4* allele. Moreover, it should be noted that in the group of APOE4/4 brain samples, only a marginal, but statistically non-significant, difference between males and females was revealed.

Altogether, the differences in brain lipidomes and transcriptomic profiles associated with *APOE* genotypes demonstrated in our study strongly support the idea that the efficiency of unfolded protein response, response to ER stress, intracellular proteasomal and lysosomal degradation, and better preserved mitochondrial function provides a molecular background for *APOE*-associated differences in AD pathology, interpreted as driven by the *APOEε2/c* group. In studies like ours, however, significant differences in “omics” profiles could raise a concern that the differences might be either due to age or AD brain pathology, including the integrity of RNA as a PMI-dependent variable. We present results based on the methodology for processing AD brain samples and statistical analyses of high-throughput datasets according to the widely accepted and rigorous standards [81]. Since the age of patients at the time of death between the groups is statistically indistinguishable (one-way ANOVA), the age as a factor, most probably, does not play a significant role. To discern whether the differences can be clearly attributed to *APOEε2* or there is a significant contribution of AD pathology is a more difficult task. The difficulties are primarily associated with the availability and thus an insufficient number of samples of *APOEε2/2* and *APOEε2/c* genotypes. The nearest consequence is that *APOEε2/c* cases are overwhelmingly of lower Braak stages, and thus, within a relatively small pool of only several hundred of AD samples, a randomized, yet homogenous group of *APOEε2/c* samples, age-matched to the other two groups—*APOEε3/3* and *APOEε4/c*—and at advanced level of AD pathology is difficult, or impossible, to construct. An alternative explanation of the demographic structure of *APOEε2/c* cases with samples predominantly in lower Braak stages would be that unlike *APOEε3/3* and *APOEε4/c*, *APOEε2/c* genotype confers genomic and likely epigenomic environment or promotes metabolic pathways that altogether have a protective effect and slow down the progression of AD and neurodegenerative pathology. The initial analysis of the *APOEε2/c* group of samples included in this study did not identify differential gene expression between the subgroups based solely on Braak stage—2, 3, and 4 vs 5 and 6 (data not shown). Since *APOEε2/c* genotype (excluding *APOEε2/4*) is consistently associated with lower Braak stages and less prominent AD brain pathology, early activation and properly functioning autophagic-lysosomal degradation, improved myelination and slower myelin breakdown might explain the better clinical outcomes observed overwhelmingly in patients of *APOEε2/c* genotype. With the relatively small sample size of the *APOEε2/c* group, intrinsic difficulties in obtaining samples at the early stages of the disease regardless of the genotype and lack of experimental designs allowing functional studies using postmortem AD brain prevent immediate testing of this hypothesis. In a study aiming at gene expression profiles differentially associated with *APOE* genotype at the time of death, there are additional limitations: for postmortem samples, age matched at the time of death and segregated by APOE genotype, the age when the cognitive decline was first recorded, and thus the duration of the disease remains unknown. It is known, however, that age is an important variable in the earlier stages of the disease, and significantly affects the progression, depending on the *APOE* genotype [7, 82] particularly if *APOEε2/c* is included in the comparisons. Finally, while we are far from understanding the role of remote mechanisms above local interactions in the evolution of AD [83], the pattern of metabolic brain alteration is likely a result of changes in the gene expression including brain areas far from MTL. Availability and transcriptomic analysis of samples of other brain areas would certainly strengthen the conclusions of a study like ours.

Despite the limitations, the results presented here support the future investigation to reveal the significance of improved myelination, more efficient autophagic-lysosomal degradation, response to ER stress, and reduced levels of intracellular toxic Tau oligomers in *APOEε2/c* individuals, ultimately slowing down the development and progression of the disease. While we still do not know if an impaired autophagic-lysosomal pathway and ER stress response, per se, is critical in prodromal AD, and how important relevant changes of the genome-wide regulatory networks are for AD progression, a systematic multi-omics approach, using postmortem AD brain samples provided by multiple AD Research Centers, will greatly facilitate the next steps towards identifying meaningful therapeutic targets.

## Conclusions

This study provides detailed transcriptomic profiling of *APOEε2/c*, *APOEε3/3*, and *APOEε4/c* postmortem brain samples of the inferior parietal lobule and demonstrates that major *APOEε2* allele-associated differences in gene expression are related to intracellular protein and organelle degradation, unfolded protein response, mitochondrial function, and posttranscriptional modifications of mRNA conducted by small non-coding RNA. The analysis of lipidomics datasets and the correlation of changes to expression levels of individual genes allow us to conclude that dysregulated expression of those involved in the control of autophagy are a characteristic for inferior parietal lobule at late stages of AD. The results of multiple analyses, within and between lipidomes and transcriptomes, also indicate that the availability of lipids and their APOE mediated transport are likely very important for the differences between the phenotypes.

## Supplementary information

**Additional file 1: Table S1.** ME membership.

**Additional file 2: Table S2.** Lipid classes, color codes & abbreviations.

**Additional file 3: Table S3.** Lipid classes, color codes & abbreviations.

**Additional file 4: Table S4.** Lipid classes, color codes & abbreviations.

**Additional file 5: Table S5.** Lipid classes, color codes & abbreviations.

**Additional file 6: Figure S1.** Gene Co-expression Network modules – correlation to sex, age and APOE genotype. WGCNA was applied to determine the correlation of Module Eigengenes (ME) to sex, age and APOE allele combinations (A) The relationship table shows the correlation between the module eigengene (rows) and sex or age (columns) with Pearson correlation values and *p*-values in parentheses. (B) The relationship table shows correlation with each APOE genotype (a more detailed presentation of this panel is provided on Figure 2). Red denotes a positive and blue denotes a negative correlation. (C) Connectivity for each module with the sex variable, depicting only one module identified with a significant correlation to sex. (D) The fraction of total genes (17572) that are assigned to each module, with only 44 genes comprising the MEsalmon. (E) Eigengene barplots for MEsalmon with each sample shown and grouped by *APOE* genotype and sex.

## Data Availability

The sequencing datasets are assembled in the required format and upon the acceptance of the manuscript for publication will be submitted and will be available from NCBI GEO.

## Abbreviations

ABCA1: ATP-binding cassette transporter A1
AD: Alzheimer’s disease
*APOE*: Apolipoprotein E
ATG: Autophagy genes
BECN1: Beclin1
CAR: Carnitine
CBS: Cerebroside
CER: Ceramide
CL: Cardiolipin
DAVID: Database for Annotation, Visualization, and Integrated Discovery
DE: Differentially expressed
ER: Endoplasmic reticulum
ERAD: ER-associated degradation
FC: Fold change
FDR: False discovery rate
GO: Gene Ontology
GS: Gene significance
LDL: Low-density lipoprotein
LPC: Lyso-phosphatidylcholine
LPE: Lyso-phosphatidylethanolamine
MCI: Mild cognitive impairment
MDMS-SL: Multidimensional mass spectrometry shotgun lipidomics
ME: Module eigengenes
MM: Module membership
MTL: Medial temporal lobe
PA: Phosphatidic acid
PC: Phosphatidylcholine
PE: Phosphatidylethanolamine
PG: Phosphatidylglycerol
PI: Phosphatidylinositol
PIP: Phosphatidylinositol phosphate
PIP2: Phosphatidylinositol bisphosphate
PIP3: Phosphatidylinositol triphosphate
PMI: Postmortem Interval
PS: Phosphatidylserine
SM: Sphingomyelin
SRP: Signal recognition particle
ST: Sulfatide
Ub: Ubiquitin
VLDL: Very low-density lipoprotein
WGCNA: Weighted gene co-expression network analysis
