# Supplementary figures and images for: APOE2 orchestrated differences in transcriptomic and lipidomic profiles of postmortem AD brain
Source: Alzheimers Res Ther. 2019 Dec 30;11:113. doi: 10.1186/s13195-019-0558-0 (PMC6937981; doi:10.1186/s13195-019-0558-0)

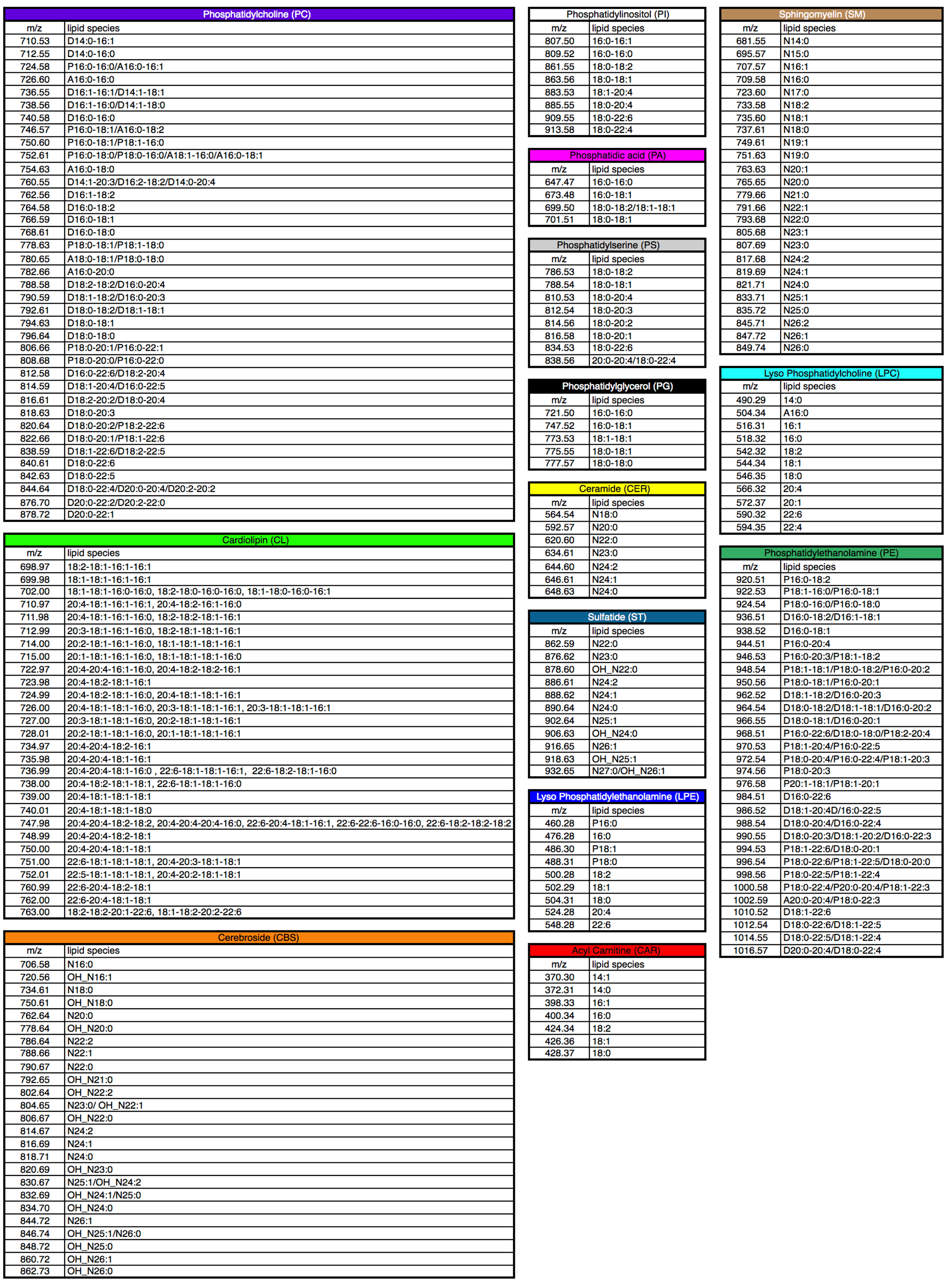

Supplement: Supplementary file 2 — Additional file 2: Table S2. Lipid classes, color codes & abbreviations. [file 13195_2019_558_MOESM2_ESM.tif]

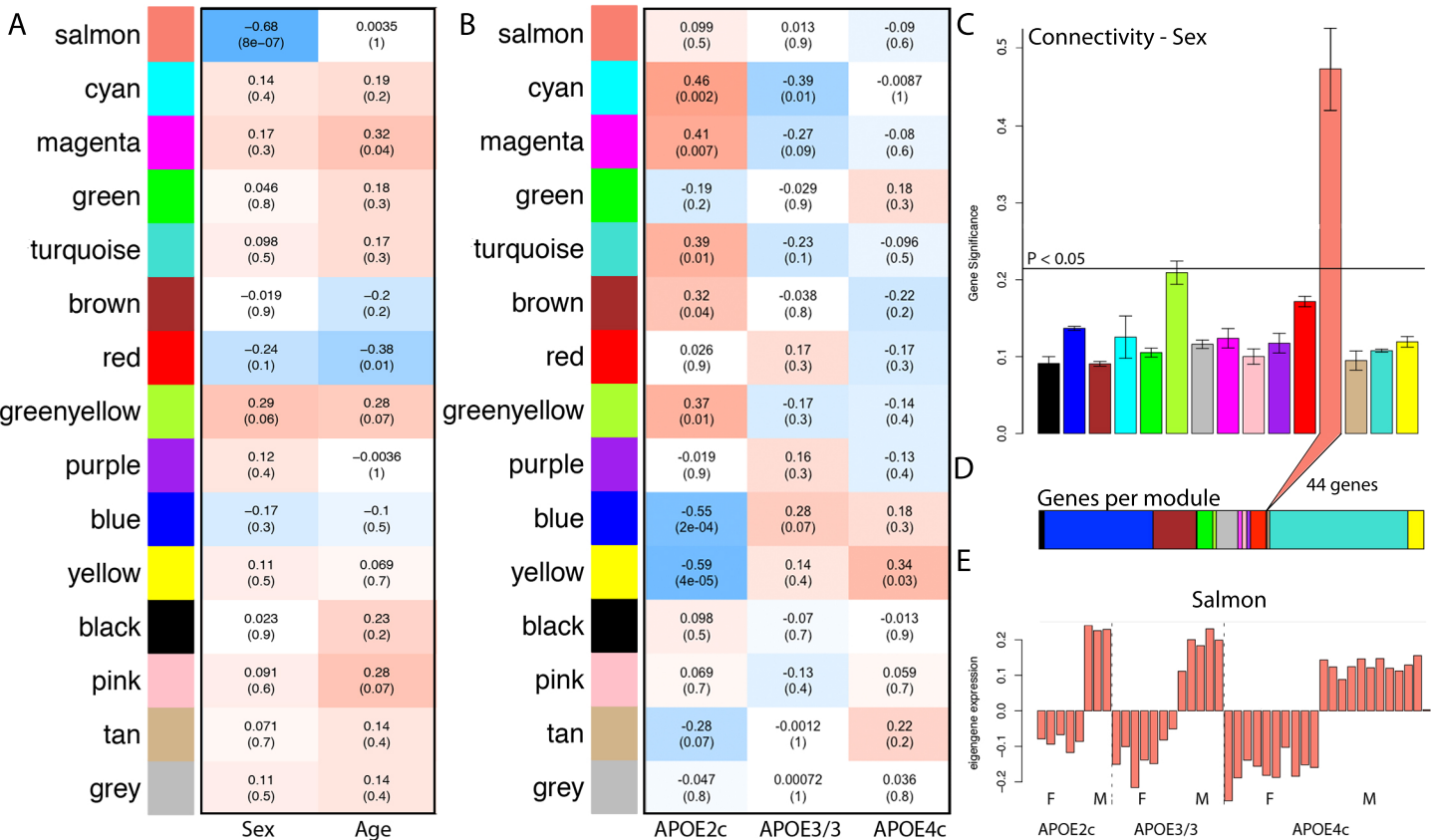

**Supplemental Figure 1**

Supplement: Supplementary file 6 — Additional file 6: Figure S1. Gene Co-expression Network modules – correlation to sex, age and APOE genotype. WGCNA was applied to determine the correlation of Module Eigengenes (ME) to sex, age and APOE allele combinations (A) The relationship table shows the correlation between the module eigengene (rows) and sex or age (columns) with Pearson correlation values and p-values in parentheses. (B) The relationship table shows correlation with each APOE genotype (a more detailed presentation of this panel is provided on Figure 2). Red denotes a positive and blue denotes a negative correlation. (C) Connectivity for each module with the sex variable, depicting only one module identified with a significant correlation to sex. (D) The fraction of total genes (17572) that are assigned to each module, with only 44 genes comprising the MEsalmon. (E) Eigengene barplots for MEsalmon with each sample shown and grouped by APOE genotype and sex. [file 13195_2019_558_MOESM6_ESM.pdf]
