# Supplementary material for: APOE2 orchestrated differences in transcriptomic and lipidomic profiles of postmortem AD brain
Source: Alzheimers Res Ther. 2019 Dec 30;11:113. doi: 10.1186/s13195-019-0558-0 (PMC6937981; doi:10.1186/s13195-019-0558-0)
Supplement: Supplementary file 3 — Additional file 3: Table S3. Lipid classes, color codes & abbreviations. [file 13195_2019_558_MOESM3_ESM.pdf]

Cardiolipin (CL)

m/z	lipid species
698.97	18:2-18:1-16:1-16:1
699.98	18:1-18:1-16:1-16:1
702.00	18:1-18:1-16:0-16:0, 18:2-18:0-16:0-16:0, 18:1-18:0-16:0-16:1
710.97	20:4-18:1-16:1-16:1, 20:4-18:2-16:1-16:0
711.98	20:4-18:1-16:1-16:0, 18:2-18:2-18:1-16:1
712.99	20:3-18:1-16:1-16:0, 18:2-18:1-18:1-16:1
714.00	20:2-18:1-16:1-16:0, 18:1-18:1-18:1-16:1
715.00	20:1-18:1-16:1-16:0, 18:1-18:1-18:1-16:0
722.97	20:4-20:4-16:1-16:0, 20:4-18:2-18:2-16:1
723.98	20:4-18:2-18:1-16:1
724.99	20:4-18:2-18:1-16:0, 20:4-18:1-18:1-16:1
726.00	20:4-18:1-18:1-16:0, 20:3-18:1-18:1-16:1, 20:3-18:1-18:1-16:1
727.00	20:3-18:1-18:1-16:0, 20:2-18:1-18:1-16:1
728.01	20:2-18:1-18:1-16:0, 20:1-18:1-18:1-16:1
734.97	20:4-20:4-18:2-16:1
735.98	20:4-20:4-18:1-16:1
736.99	20:4-20:4-18:1-16:0 , 22:6-18:1-18:1-16:1, 22:6-18:2-18:1-16:0
738.00	20:4-18:2-18:1-18:1, 22:6-18:1-18:1-16:0
739.00	20:4-18:1-18:1-18:1
740.01	20:4-18:1-18:1-18:0
747.98	20:4-20:4-18:2-18:2, 20:4-20:4-20:4-16:0, 22:6-20:4-18:1-16:1, 22:6-22:6-16:0-16:0, 22:6-18:2-18:[illegible]
748.99	20:4-20:4-18:2-18:1
750.00	20:4-20:4-18:1-18:1
751.00	22:6-18:1-18:1-18:1, 20:4-20:3-18:1-18:1
752.01	22:5-18:1-18:1-18:1, 20:4-20:2-18:1-18:1
760.99	22:6-20:4-18:2-18:1
762.00	22:6-20:4-18:1-18:1
763.00	18:2-18:2-20:1-22:6, 18:1-18:2-20:2-22:6

Lyso Phosphatidylethanolamine (LPE)

m/z	lipid species
460.28	P16:0
476.28	16:0
486.30	P18:1
488.31	P18:0
500.28	18:2
502.29	18:1
504.31	18:0
524.28	20:4
548.28	22:6

Acyl Carnitine (CAR)

m/z	lipid species
370.30	14:1
372.31	14:0
398.33	16:1
400.34	16:0
424.34	18:2
426.36	18:1
428.37	18:0

Ceramide (CER)

m/z	lipid species
564.54	N18:0
592.57	N20:0
620.60	N22:0
634.61	N23:0
644.60	N24:2
646.61	N24:1
648.63	N24:0

Suppl Table 3 - Lipids
