# Supplementary material for: APOE2 orchestrated differences in transcriptomic and lipidomic profiles of postmortem AD brain
Source: Alzheimers Res Ther. 2019 Dec 30;11:113. doi: 10.1186/s13195-019-0558-0 (PMC6937981; doi:10.1186/s13195-019-0558-0)
Supplement: Supplementary file 4 — Additional file 4: Table S4. Lipid classes, color codes & abbreviations. [file 13195_2019_558_MOESM4_ESM.pdf]

Phosphatidylcholine (PC)	
m/z	lipid species
710.53	D14:0-16:1
712.55	D14:0-16:0
724.58	P16:0-16:0/A16:0-16:1
726.60	A16:0-16:0
736.55	D16:1-16:1/D14:1-18:1
738.56	D16:1-16:0/D14:1-18:0
740.58	D16:0-16:0
746.57	P16:0-18:1/A16:0-18:2
750.60	P16:0-18:1/P18:1-16:0
752.61	P16:0-18:0/P18:0-16:0/A18:1-16:0/A16:0-18:1
754.63	A16:0-18:0
760.55	D14:1-20:3/D16:2-18:2/D14:0-20:4
762.56	D16:1-18:2
764.58	D16:0-18:2
766.59	D16:0-18:1
768.61	D16:0-18:0
778.63	P18:0-18:1/P18:1-18:0
780.65	A18:0-18:1/P18:0-18:0
782.66	A16:0-20:0
788.58	D18:2-18:2/D16:0-20:4
790.59	D18:1-18:2/D16:0-20:3
792.61	D18:0-18:2/D18:1-18:1
794.63	D18:0-18:1
796.64	D18:0-18:0
806.66	P18:0-20:1/P16:0-22:1
808.68	P18:0-20:0/P16:0-22:0
812.58	D16:0-22:6/D18:2-20:4
814.59	D18:1-20:4/D16:0-22:5
816.61	D18:2-20:2/D18:0-20:4
818.63	D18:0-20:3
820.64	D18:0-20:2/P18:2-22:6
822.66	D18:0-20:1/P18:1-22:6
838.59	D18:1-22:6/D18:2-22:5
840.61	D18:0-22:6
842.63	D18:0-22:5
844.64	D18:0-22:4/D20:0-20:4/D20:2-20:2
876.70	D20:0-22:2/D20:2-22:0
878.72	D20:0-22:1

Phosphatidylinositol (PI)	
m/z	lipid species
807.50	16:0-16:1
809.52	16:0-16:0
861.55	18:0-18:2
863.56	18:0-18:1
883.53	18:1-20:4
885.55	18:0-20:4
909.55	18:0-22:6
913.58	18:0-22:4

Phosphatidic acid (PA)	
m/z	lipid species
647.47	16:0-16:0
673.48	16:0-18:1
699.50	18:0-18:2/18:1-18:1
701.51	18:0-18:1

Phosphatidylserine (PS)	
m/z	lipid species
786.53	18:0-18:2
788.54	18:0-18:1
810.53	18:0-20:4
812.54	18:0-20:3
814.56	18:0-20:2
816.58	18:0-20:1
834.53	18:0-22:6
838.56	20:0-20:4/18:0-22:4

Phosphatidylglycerol (PG)	
m/z	lipid species
721.50	16:0-16:0
747.52	16:0-18:1
773.53	18:1-18:1
775.55	18:0-18:1
777.57	18:0-18:0

Suppl Table 4 - Lipids
