# Supplementary material for: APOE2 orchestrated differences in transcriptomic and lipidomic profiles of postmortem AD brain
Source: Alzheimers Res Ther. 2019 Dec 30;11:113. doi: 10.1186/s13195-019-0558-0 (PMC6937981; doi:10.1186/s13195-019-0558-0)
Supplement: Supplementary file 5 — Additional file 5: Table S5. Lipid classes, color codes & abbreviations. [file 13195_2019_558_MOESM5_ESM.pdf]

Phosphatidylethanolamine (PE)	
m/z	lipid species
920.51	P16:0-18:2
922.53	P18:1-16:0/P16:0-18:1
924.54	P18:0-16:0/P16:0-18:0
936.51	D16:0-18:2/D16:1-18:1
938.52	D16:0-18:1
944.51	P16:0-20:4
946.53	P16:0-20:3/P18:1-18:2
948.54	P18:1-18:1/P18:0-18:2/P16:0-20:2
950.56	P18:0-18:1/P16:0-20:1
962.52	D18:1-18:2/D16:0-20:3
964.54	D18:0-18:2/D18:1-18:1/D16:0-20:2
966.55	D18:0-18:1/D16:0-20:1
968.51	P16:0-22:6/D18:0-18:0/P18:2-20:4
970.53	P18:1-20:4/P16:0-22:5
972.54	P18:0-20:4/P16:0-22:4/P18:1-20:3
974.56	P18:0-20:3
976.58	P20:1-18:1/P18:1-20:1
984.51	D16:0-22:6
986.52	D18:1-20:4D/16:0-22:5
988.54	D18:0-20:4/D16:0-22:4
990.55	D18:0-20:3/D18:1-20:2/D16:0-22:3
994.53	P18:1-22:6/D18:0-20:1
996.54	P18:0-22:6/P18:1-22:5/D18:0-20:0
998.56	P18:0-22:5/P18:1-22:4
1000.58	P18:0-22:4/P20:0-20:4/P18:1-22:3
1002.59	A20:0-20:4/P18:0-22:3
1010.52	D18:1-22:6
1012.54	D18:0-22:6/D18:1-22:5
1014.55	D18:0-22:5/D18:1-22:4
1016.57	D20:0-20:4/D18:0-22:4

Suppl Table 5 - Lipids
